# Enhancing human-induced pluripotent stem cell proliferation and cardiac differentiation through 810-nm photobiomodulation

**DOI:** 10.1007/s10103-026-04821-8

**Published:** 2026-02-10

**Authors:** Wei-Zhen Kao, Huai-Ching Hsieh, Yi-Ju Lee, Jin-Yu Su, Pin-Yun Shen, An-Chi Wei

**Affiliations:** National Taiwan University, Taipei, Taiwan

**Keywords:** Photobiomodulation, 810 nm LED, iPSC, Differentiation

## Abstract

Photobiomodulation (PBM) has been shown to enhance cell growth and differentiation. This study investigates the effects of 810-nm PBM on the proliferation and cardiac differentiation of human-induced pluripotent stem cells (hiPSCs), utilizing a custom-made LED device. This device is compatible with standard cell incubators and features an LED array that ensures uniform light exposure to cells in each well of a 96-well plate. hiPSCs were irradiated with 810-nm light at intensities of 1.0, 1.5, and 2.0 mW/cm² for 15 min daily. Outcomes assessed included cell viability, mitochondrial function and morphology, and cardiomyocyte differentiation. The results show that PBM at 1.5 mW/cm² enhanced hiPSC proliferation and cardiac differentiation, yielding a higher number of spontaneously beating cardiomyocytes compared to controls. These findings suggest that 810-nm PBM may improve the efficiency of cardiac cell generation for both research and therapeutic applications.

**Supplementary Information:**

The online version contains supplementary material available at 10.1007/s10103-026-04821-8.

## Introduction

Human induced pluripotent stem cells (hiPSCs) have emerged as a cornerstone in regenerative medicine and disease modeling due to their ability to differentiate into various cell types. Among these, iPSC-derived cardiomyocytes (iPSC-CMs) are now widely used as a model system for studying cardiovascular development, disease mechanisms, and drug screening [1]. The ability to generate patient-specific iPSC-CMs has also facilitated personalized medicine in cardiovascular research [2]. However, the differentiation of hiPSCs into cardiomyocytes remains challenging due to the lengthy process, low efficiency, and difficulty in achieving fully mature and functional cardiac cells that are capable of recapitulating the physiological properties of native cardiomyocytes [3, 4]. Photobiomodulation (PBM) is an increasingly popular technique that uses low-energy red or near-infrared light to stimulate biological tissues with applications in both biomedical and clinical research. Studies have demonstrated the ability of PBM to increase cell growth and proliferation across different cell types [5–9]. Furthermore, PBM has shown promise in promoting stem cell differentiation, with specific ranges of light intensity and wavelengths proving effective in promoting lineage commitment [7]. For example, PBM was reported to facilitate osteogenic differentiation in mesenchymal stem cells and neurogenic differentiation in neural progenitor cells [10]. Notably, Atum et al. demonstrated that PBM can mitigate doxorubicin-induced cardiotoxicity in hiPSC-derived cardiomyocytes [11], further highlighting PBM’s potential in cardiovascular applications.

Despite these advancements, the understanding of the mechanisms and effects of PBM on hiPSC growth and cardiac differentiation remains limited. In this study, we utilized a custom-designed, incubator-compatible LED device to study the long-term irradiation effects on hiPSCs. The device setup enabled precise control of light irradiation without perturbing the cell culture environment. We investigated the effects of PBM on cell viability and cardiac differentiation to evaluate the potential use and the underlying mechanisms of PBM in hiPSCs. Our findings demonstrate that PBM enhances hiPSC differentiation efficiency and improves the functionality of iPSC-CMs, supporting their potential for therapeutic applications.

## Methods

### Cell culture and cardiomyocyte differentiation

Human induced pluripotent stem cells (hiPSCs) (Coriell AICS-0010) were obtained and utilized in this study. The hiPSCs were cultured according to the manufacturer’s protocol (Culture and Freezing Methods for WTC Derived AICS hiPSC Lines, Allen Institute for Cell Science). The cells were grown on dishes precoated with 0.337 mg/mL Matrigel and in 0.22-µm-filtered mTeSR1 medium with 1% penicillin/streptomycin and 10 mM ROCK inhibitor at a 1:1000 dilution on the first day of passage at 37 °C and 5% CO_2_ for 24 h. After 24 h, the medium was changed to 0.22-µm-filtered mTeSR1 medium supplemented with 1% penicillin/streptomycin.

**Fig. 1:**
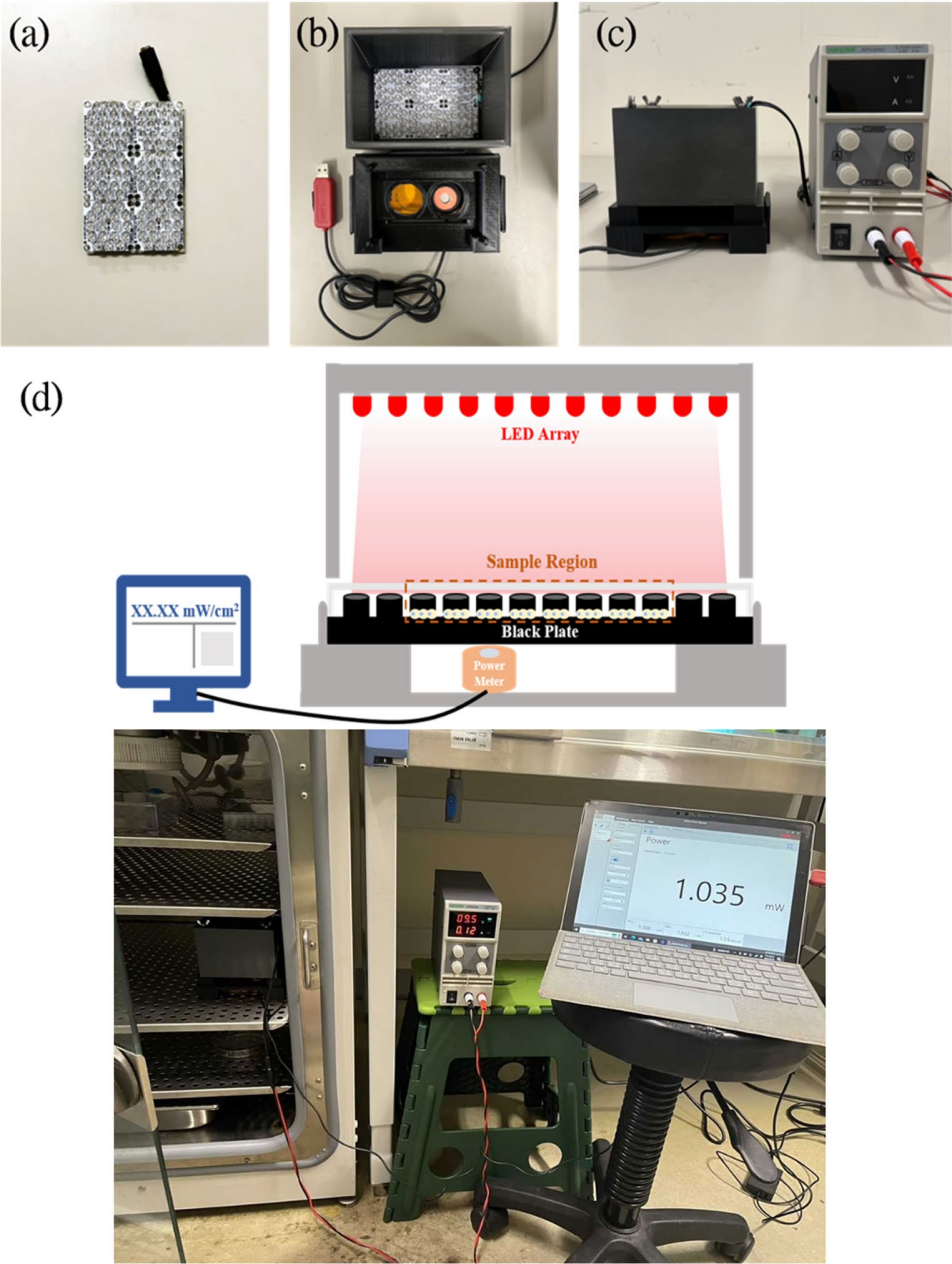
Custom-made 810 nm LED device for this PBM study. (**a**) 810 nm LED array; (**b**) at the top is the lid combined with the LED, and at the bottom is the 3D printed platform and power meter; (**c**) the microincubator with the LED array connected to the power supply; (**d**) the overview of the PBM device setup

After the confluence of the seeded hiPSCs reached approximately 70 ~ 90%, the medium was changed to RPMI/B27(-) medium supplemented with 7.5 µM CHIR99021, and this day was considered Day 0 of the differentiation process. On Day 2, the medium was changed to RPMI/B27(-) medium with 7.5 µM IWP2. Then, RPMI medium was added on Day 4. After 2 days, the medium was changed to RPMI/B27(+), and fresh RPMI/B27(+) medium was continuously changed every 2 days until Day 14 (Fig. 1). After Day 14, the medium was changed twice per week. The primary differentiation vehicles used were 35 mm confocal dishes (1500 µL of medium) and black 96-well plates (100 µL of medium per well) [12, 13].

### Setup of the photombioodulation device

To ensure precise execution of the light treatment, we developed and utilized a custom-designed device for the PBM. An array of 810 nm LEDs ( KOODYZ Technology Co., Taiwan) were aligned along with a lid manufactured through 3D printing and secured in place with screws and nuts, completing our primary light irradiation setup. With the combination of a power supply and a custom-made platform, we established our light irradiation system. To ensure accuracy in light delivery, a power meter was positioned beneath the platform to continuously monitor light irradiance. The readings were concurrently displayed on a central computer, enabling real-time adjustments. This setup allowed us to maintain irradiance within an error range of ± 0.05 mW/cm², ensuring consistent and reliable treatment conditions. Figure 1 shows the entire system setup.

### Parameters and conditions of photobiomodulation

**Fig. 2:**
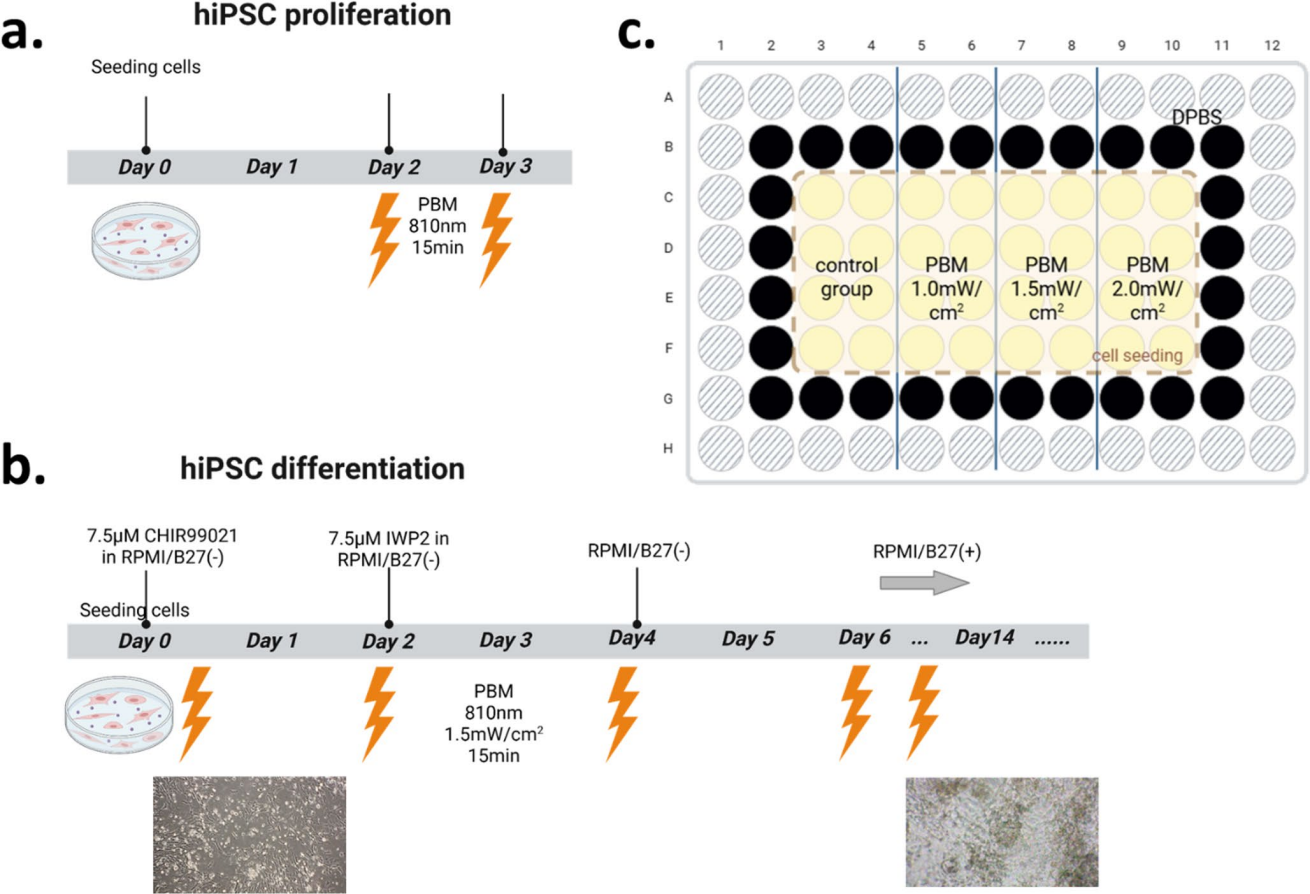
Experimental PBM protocols and plate layout. (**a**, **b**) Schematic representation of the photobiomodulation (PBM) protocols used for hiPSC proliferation (**a**) and differentiation (**b**). (**c**) Layout of the 96-well plate used for intensity optimization. A total of 32 wells were divided into four experimental groups: a non-irradiated Control and three groups exposed to 810 nm light at irradiances of 1.0, 1.5, and 2.0 mW/cm². For the hiPSC differentiation experiments, 1.5 mW/cm^2^ was used for 15 min of light irradiation treatments every 2 days. The figure was created by Biorender.com

For undifferentiated cells, 2 days after subculture, the hiPSCs were irradiated with an 810 nm LED in a custom-made microincubator once a day for 2 days (Fig. 2a). The cell culture plate was placed in the microincubator only during the 15-minute light treatment. For the remainder of the time, the plate was maintained in a regular incubator at 37 °C and 5% CO_2_. Based on the accepted power density used in PBM, we tested three different doses on the sample [7, 9]. The 96-well black plates were used to avoid possible inter-well light interference (Fig. 2c). To ensure consistent irradiation, only the central 32 wells of a 96-well plate were seeded with cells, and Dulbecco’s phosphate-buffered saline (DPBS) was applied to the border wells to prevent evaporation of the culture medium from these wells.

For hiPSC-derived cardiomyocytes, we used 1.5 mW/cm^2^ as the intensity dosage for further differentiation experiments on the basis of our findings in 810 nm light-treated undifferentiated cells. In addition, 15-minute light treatments were performed every 2 days, after the differentiation medium was changed [10](Fig. 2b).

### Cell viability assay

Cell viability was assessed with a Cell Counting Kit-8 (CCK-8) (MedChemExpress, USA). iPSCs (5500 cells per well) were first seeded in a black 96-well plate. Twenty-four hours after seeding, the medium was replaced with mTeSR1 medium. The first 15-min light treatment was executed following the next medium change (i.e., 48 h after cell seeding). After 2 days of light treatment, cell viability assays were performed [9] and the results were measured with a SpectraMax iD3 plate reader (Molecular Devices, USA).

### Cell cycle analysis

The cell samples were fixed with 9:1 70% ice-cold ethanol and DPBS at -20 °C for 1 h and stored at 4 °C overnight [9]. Fixed cells were then stained with 50 µg/mL propidium iodide (PI; Thermo Fisher Scientific, USA) and 100 µg/mL RNase A (Thermo Fisher Scientific, USA) in DPBS solution to facilitate DNA content analysis. Flow cytometric analysis was performed using an LSRFortessa flow cytometer (BD Biosciences, USA). ModFit LT 5.0 was used to analyze the histograms of PI signals and cell cycle phases.

### Cellular oxidative stress assay

The reactive oxygen species assay was evaluated similarly to the cell viability assay. Black 96-well plates were seeded with 5500 cells in each well. Fifteen minutes of light treatment daily began after 48 h of seeding. After 2 days of light treatment, the reactive oxygen species were measured using the CellROX DeepRed Reagent (Thermo Fisher, USA), and the absorbance was measured by a SpectraMax iD3 plate reader (Molecular Devices, USA).

### Cellular respiration analysis using high-resolution respirometry

Oxygen consumption rates (OCRs) were assessed using a high-resolution Oxygraph-2k respirometer (O2k, Oroboros Instruments, Innsbruck, Austria) [14]. Following harvesting, undifferentiated iPSCs were suspended in mTeSR1 medium at a concentration of 10^6^ cells/mL. The oxygen measurements were carried out in a closed system at 37 °C with a stirring rate of 500 rpm. Basal respiration (or routine respiration) was first recorded without the addition of mitochondrial inhibitors (in mTeSR1). To assess respiration in the leak state (a nonphosphorylating resting state), oligomycin (ATP synthase inhibitor) was introduced. Subsequently, stepwise titration of carbonyl cyanide-p-trifluoromethoxyphenylhydrazone (FCCP; a mitochondrial uncoupler) was performed to determine the maximal capacity of the mitochondrial electron transport system. Rotenone (complex I inhibitor) and antimycin A (complex III inhibitors) were then added to measure the OCR in the ROX state (a state with residual respiration) [9].

### Western blot analysis

To evaluate the relative protein levels in cardiomyocytes, total protein was extracted using RIPA lysis and extraction buffer (Thermo Fisher Scientific, USA). The extracted proteins were then denatured and loaded on Q-PAGETM TGN Precast Gel (Smobio, Taiwan). Following electrophoretic separation, the proteins were transferred to a polyvinylidene difluoride (PVDF) membrane by electroblotting. Primary antibodies against myosin light chain 7 (MYL7) (ab127001, Abcam), troponin T (TnT) (ab209813, Abcam), myosin light chain 2 (MYL2) (MA5-15496, Thermo Fisher), Cyclin D1 (CCND) (Ab134175, Abcam) and Total OXPHOS Human WB Antibody Cocktail (1:800, Ab110411 Abcam) were used for cardiomyocyte and mitochondria characterization. Anti-beta actin antibody (ab8226, Abcam, U.K.) was used as a loading control. Horseradish peroxidase (HRP)-conjugated goat-anti-mouse IgG (ab97023, Abcam, U.K.) was used as the secondary antibody. Protein detection was performed using enhanced chemiluminescence (ECL) substrates (Thermo Fisher Scientific, USA) and chemiluminescent signals were captured by a UVP ChemStudio Imaging System (Analytik Jena, Germany) with an exposure time of 30 s for level analysis in Image Lab 6.1 (Bio-Rad, USA) [15].

### Confocal fluorescence imaging and analysis

For imaging purposes, the iPSCs were replated on the 18th day of differentiation. In accordance with the protocol of Koc et al. [16], hiPSC-derived cardiomyocytes were washed with DPBS and 50 uL of Accutase was added to each well. After incubation for 15 min at 37 °C, 100 µL of Enzyme Inactivation Medium (0.22-µm filtered 5% FBS in RPMI 1640 Medium) was added to neutralize the enzyme. The cells were then collected and centrifuged at 300 × g for 3 min, after which the supernatant was discarded. The cell pellet was resuspended in 2 mL of RPMI/B27(+) medium containing 5 µM ROCK inhibitor and replated onto precoated confocal dishes for subsequent imaging.

On the 20th day of differentiation, hiPSC-derived cardiomyocytes were stained with MitoTracker Deep Red (Thermo Fisher Scientific, USA) to label mitochondria and with Hoechst (Thermo Fisher Scientific, USA) to stain nuclear DNA. Fluorescence images were acquired using a Zeiss LSM 800 confocal fluorescence microscope (Carl Zeiss, Germany), an Airyscan module and a 63x oil immersion objective (1.4 NA).

Mitochondrial morphology was further analyzed using the image analysis pipeline based on Hsieh et al.^9^ The individual cells were manually cropped from the confocal microscopy images. Segmentation and skeletonization of the mitochondrial images were then performed [17]. Particle features (e.g., mitochondrial counts, areas, perimeters, and solidity) and skeleton features (e.g., number and length of branches, node degree, or number of branches connecting to a node) were extracted [18]. The Skan package (version 0.10.0) in Python (version 3.8.5) was used to analyze the skeletonized images of the mitochondria.

### Quantification of cardiomyocyte beating frequency

Time-lapse imaging was performed to analyze the contraction kinetics of iPSC-CMs [19]. Videos were acquired using a confocal microscope equipped with a 10× objective in phase-contrast mode to capture pixel intensity fluctuations associated with cell contraction. Recording sessions lasted for 30 s (Day 15) and 3 min (Day 19) with a sampling frequency of 0.5-second intervals. For data extraction, the Fiji (ImageJ) software ROI Manager was used to manually select ten regions of interest (ROIs) per well. ROIs were placed at the edges of beating areas where pixel contrast variance was maximal. The mean gray value (mean pixel intensity) for each ROI was measured across all time frames to generate a time-series signal of contractile motion. To quantify beating frequency, raw intensity signals were processed using a custom Python script utilizing the scipy.signal.find_peaks function. First, signals underwent baseline adjustment and normalization via a weighted moving average to eliminate low-frequency drift artifacts caused by sample movement. Beating frequency, expressed as beats per minute (BPM), was then calculated by directly counting the detected peaks in the normalized signal. This peak-counting approach was validated against Fast Fourier Transform (FFT) analysis and selected for handling irregular beating signals in our experimental conditions.

### Statistical analysis

Data are presented as the mean ± standard deviation (SD) unless otherwise specified. Experiments were performed with a minimum of three independent biological replicates (*n* = 3). Statistical comparisons between two groups (e.g., Control vs. Light) were analyzed using an unpaired two-tailed Student’s *t*-test. To evaluate phenotypic heterogeneity in cardiomyocyte beating frequency, an F-test for equality of variances was used to assess differences in the width of data distribution. A *p*-value of < 0.05 was considered statistically significant. Statistical analyses and graph generation were performed using GraphPad Prism 10 or Python SciPy.

## Results

**Fig. 3:**
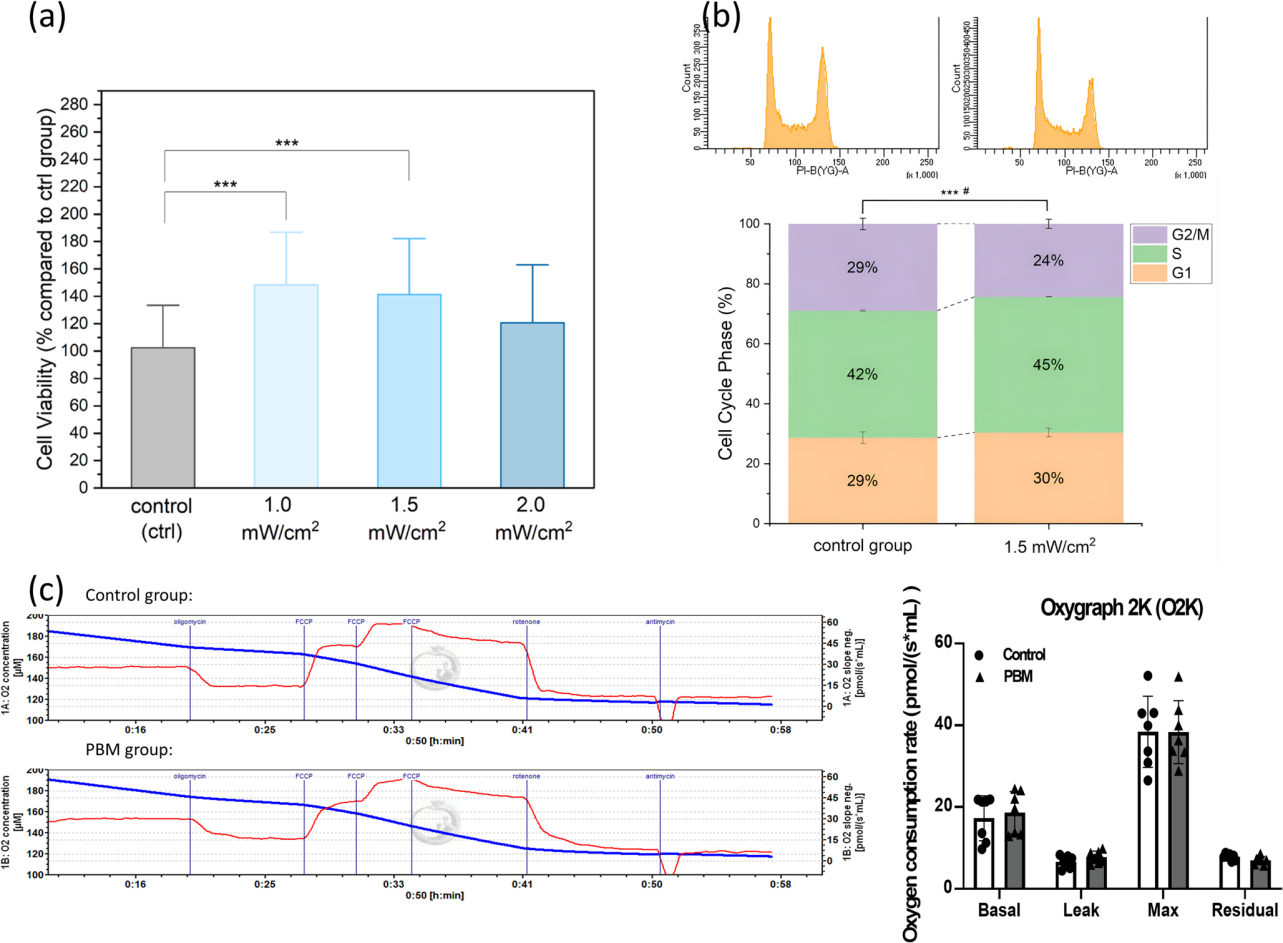
Photobiomodulation on undifferentiated iPSCs (**a**) Cell viability of undifferentiated iPSCs after light treatments. Cell viability assays were conducted after two days of daily 15-minute light treatments at intensities of 0, 1.0, 1.5, and 2.0 mW/cm². Three asterisks indicate a statistically significant difference (*p* < 0.001, *n* = 28 for each group, with each data point representing cells from a single well of a 96-well plate). (**b**) Cell cycle analysis. Representative histograms depict propidium iodide (PI) intensity in flow cytometry analysis for the control group and 1.5 mW/cm^2^ treated groups. The data are based on three independent experiments (*n* = 3), with each data point representing the total number of cells from eight wells of a 96-well plate. A statistically significant difference between the control and light-treated groups in the S-phase is indicated by three asterisks (***) (*p* < 0.001), and the G2/M phase is marked by a hashtag (#) (*p* < 0.05). (**c**) O2K oxygraph measurements of the control group and the PBM group (1.5 mW/cm^2^). The chemicals oligomycin (5µM), FCCP (5 µM), rotenone (100 µM), and antimycin A (5 µM) were added sequentially to evaluate the oxygen consumption rates (OCRs) at different bioenergetic states. Basal respiration, proton leak, maximal respiration, and residual respiration (non-mitochondrial respiration) were calculated based on chemical interventions. The average oxygen consumption rates were calculated every 45 data points in the stable regions of each state

To determine the optimal range of irradiance intensity for promoting cell proliferation, the central 32 wells of a 96-well plate were divided into four groups: a control group, and three treatment groups exposed to 810-nm light at intensities of 1.0 mW/cm², 1.5 mW/cm², and 2.0 mW/cm² for 15 min daily (Fig. 2c). After 2 days of treatment, a significant increase in cell viability was observed in the 1.0 and 1.5 mW/cm^2^ groups compared with the control group (Fig. 3a).

Based on cell viability studies with various light intensities, we chose 1.5 mW/cm^2^ as the optimum light intensity for studying the photobiomodulation effect on iPSC proliferation. The cell cycle analysis showed that the percentage of S-phase cells increased, indicating a greater proportion of actively dividing cells in the population (Fig. 3b). These findings suggested that photobiomodulation enhanced iPSCs division and proliferation, which was consistent with the cell viability results.

Given that mitochondria are recognized as the primary site of light absorption in mammalian cells, respiration rates in three different bioenergetic states were evaluated for both the control and 1.5 mW/cm² groups using high-resolution respirometry(Fig. 3c). The results showed a slight increase in average basal respiration in the 1.5 mW/cm² light-treated group, although this increase was not statistically significant.

**Fig. 4:**
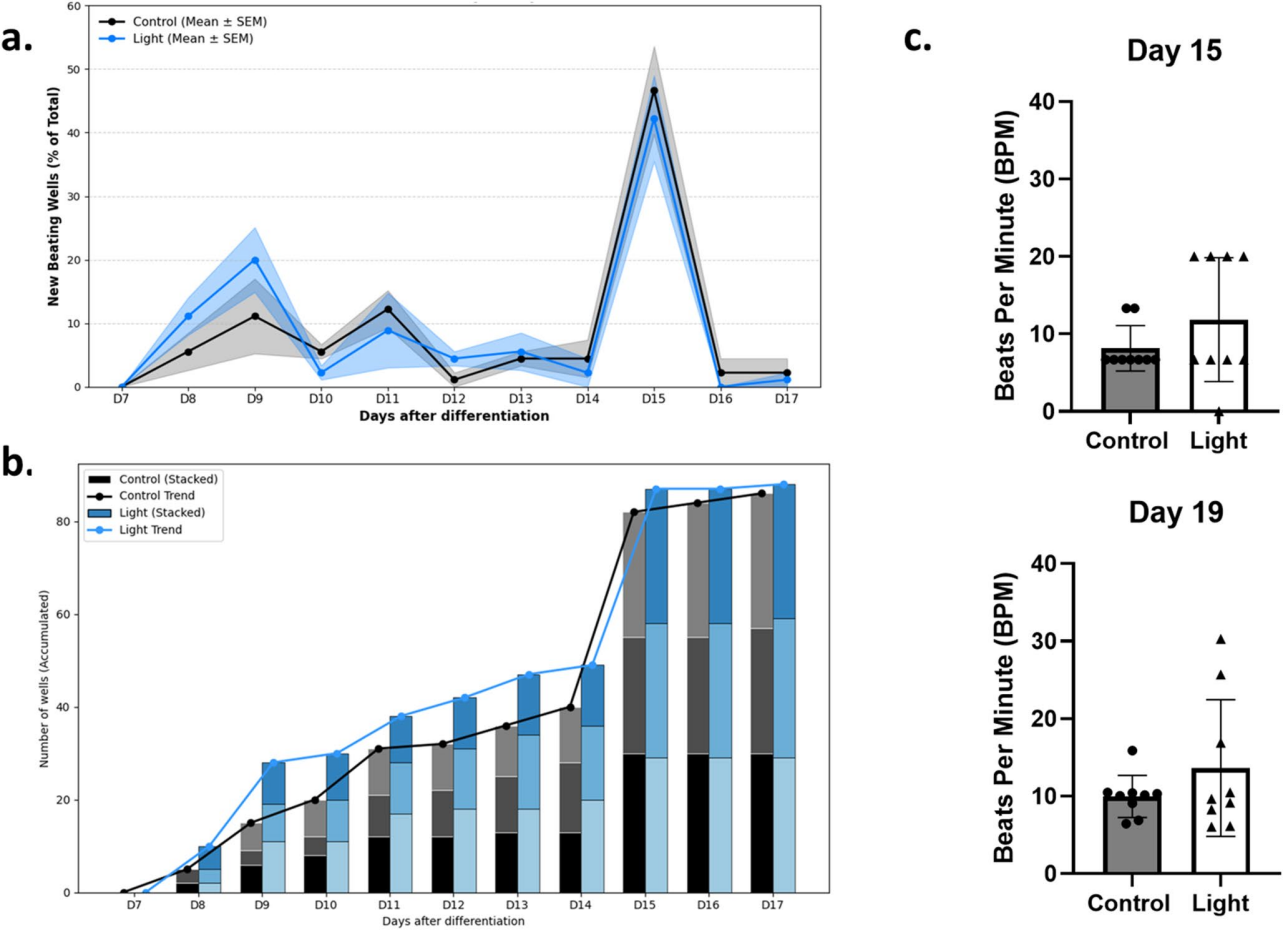
Light stimulation enhances the efficiency and kinetics of cardiomyocyte differentiation. (**a**) Daily quantification of wells exhibiting new onset of beating activity, expressed as a percentage of total seeded wells ( 30 wells per replicate). Data are presented as mean ± SEM of three independent biological replicates. Shaded bands represent the standard error of the mean (SEM). (**b**) Cumulative number of beating wells over time. Stacked bar graph showing the accumulated total of beating wells for Control (black) and Light (blue) groups from Day 7 to Day 17 post-differentiation. Data represents the sum of three independent biological replicates (3 plates), with individual replicate contributions distinguished by shading intensity. The solid trend lines (black for Control, light blue for Light) trace the total number of functioning wells across the time course. (**c**) Cardiomyocyte beating frequency at Day 15 and Day 19 post-differentiation.

Based on the findings from undifferentiated iPSCs, we maintained 1.5 mW/cm² as the irradiation intensity for subsequent differentiation experiments. Light irradiation was applied for 15 min every two days following medium changes [10]. hiPSCs were seeded in 96-well plates, and the beating activity in each well was monitored beginning on Day 7 of the differentiation process. To evaluate the impact of photobiomodulation (PBM) on cardiac differentiation efficiency, we monitored the daily onset, cumulative yield, and beating frequency of functional cardiomyocytes (Fig. 4). The light-treated group exhibited accelerated differentiation kinetics, with the first beating clusters appearing significantly earlier (Day 8–10) compared to the Control group (Fig. 4a). This kinetic advantage translated into a higher daily efficiency, peaking at Day 15 where approximately 42% of light-treated wells initiated beating. This enhancement was further reflected in the cumulative yield; the stacked bar graph analysis (Fig. 4b) confirmed that PBM treatment resulted in a consistently higher total number of functioning cardiomyocyte clusters from Day 7 through Day 17 across three independent biological replicates. Finally, to assess functional maturity, we analyzed the beating frequency of the generated cardiomyocytes (Fig. 4c). While the mean beating rate was comparable between groups, PBM treatment induced an increase in phenotypic heterogeneity (Fig. 4c). Analysis of variance revealed that by Day 19, the beating frequency in the light-treated group was more variable than in the Control group. This suggests that rather than uniformly accelerating all cells, light stimulation might promote the emergence of a sub-population of responder cardiomyocytes with high-frequency beating characteristics.

**Fig. 5:**
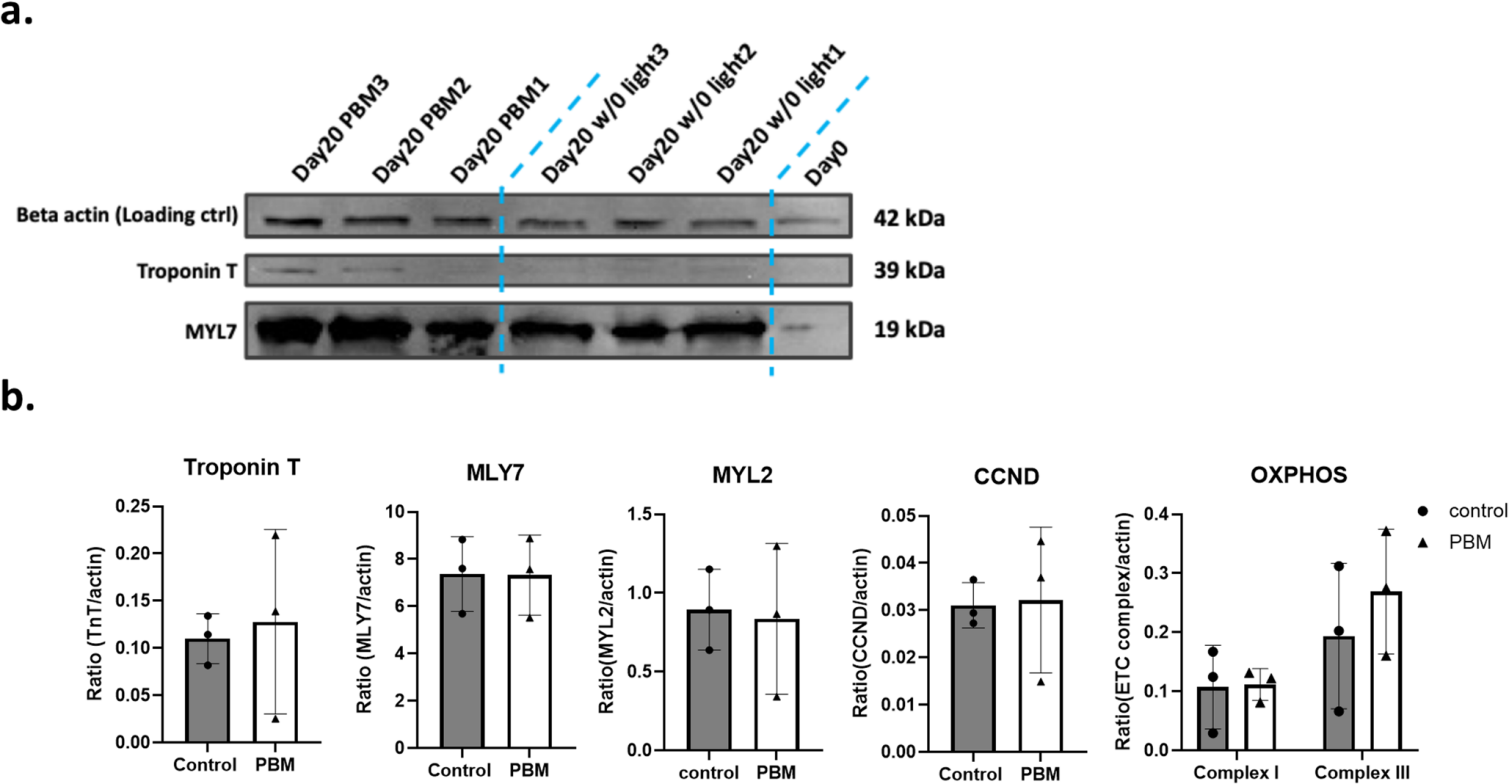
Assessment of cardiac differentiation markers in hiPSC-derived cardiomyocytes after Day 14 of differentiation. (**a**) Representative Western blot images and quantitative analysis of MYL7 and troponin T levels in control and light treatment groups on Day 20 of differentiation. (**b**) Quantitative densitometric analysis of Western blot data. Bar graphs display the relative protein expression normalized to Beta-actin for troponin T, MYL7, MYL2, CCND, and OXPHOS complexes (Complex I and Complex III) after Day 14. Beta-actin was utilized as a loading control

After Day 14 of differentiation, protein samples were collected for Western blot analysis to evaluate MYL2, MYL7 and troponin T levels as well as CCND and mitochondrial complex I and III. These biomarkers were used to assess cardiomyocyte specificity, cell cycle and mitochondrial bioenergetics. Quantitative analysis revealed slight increases in troponin T and mitochondrial complex III levels in the light-treated group compared to the control group, though these differences were not statistically significant (Fig. 5).

**Fig. 6:**
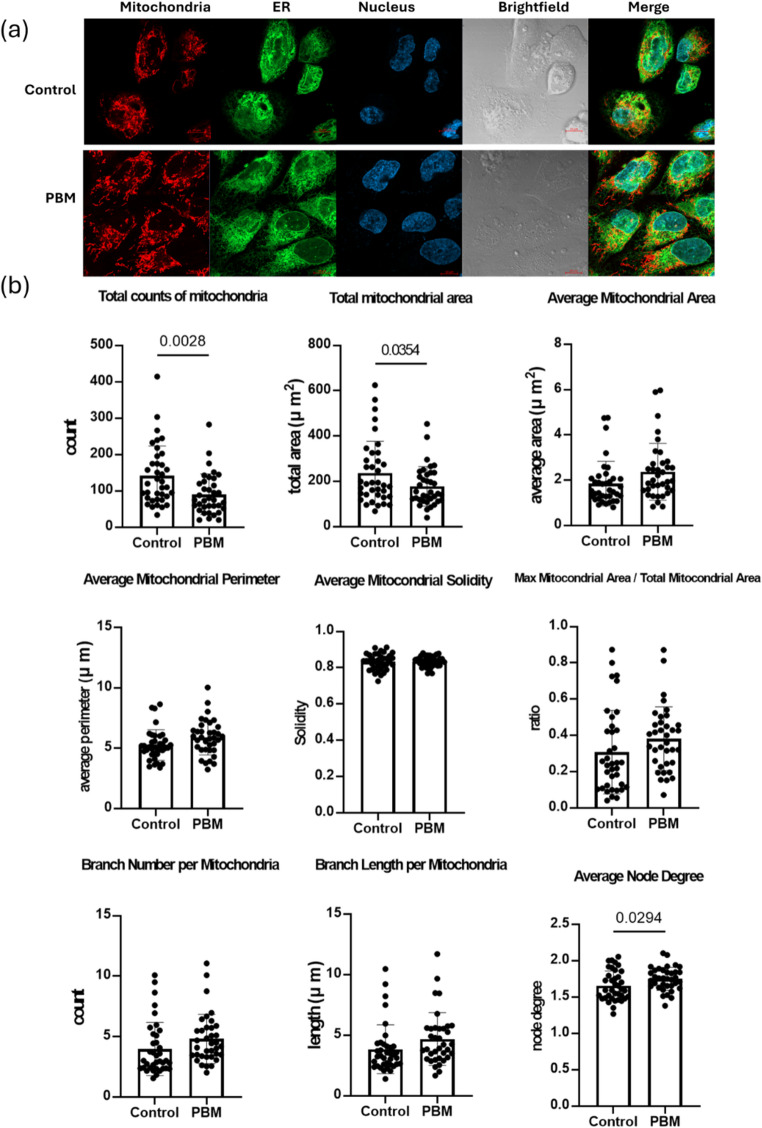
Mitochondrial morphology analysis of iPSCs. (**a**) Confocal fluorescence microscopy images and bright field images of differentiated iPSCs with and without 1.5 mW/cm^2^ light treatment (the PBM group and control group). The organelles were labeled as follows: mitochondria (red, MitoTracker Red), ER (green, mEGFP-tagged) and nucleus (blue, Hoechst). (**b**) Mitochondrial network features were extracted from microscopy images of mitochondria, with each data point representing a single cell (control group: 38 cells; 1.5 mW/cm^2^ group: 36 cells)

Mitochondrial morphology can undergo changes during stem cell differentiation. To investigate whether PBM treatment influences mitochondrial structure in hiPSC-derived cardiomyocytes, we conducted confocal microscopy analysis. On Day 18 of differentiation, cells were dissociated, replated onto confocal dishes, and stained with Hoechst and MitoTracker Red after 24 h (Fig. 6a). Quantitative assessment revealed that cardiomyocytes exposed to 1.5 mW/cm² PBM exhibited reductions in total mitochondrial count and area, accompanied by an increase in average node degree compared with controls (Fig. 6b). These findings indicate that PBM promotes reorganization of the mitochondrial network, resulting in increased network connectivity despite reduced overall mitochondrial size. To further evaluate mitochondrial functional integrity, we assessed mitochondrial membrane potential using tetramethylrhodamine methyl ester (TMRM) staining (Supplementary Fig. S2). Quantitative analysis showed no significant differences in TMRM fluorescence intensity between PBM-treated and control groups, indicating that mitochondrial membrane potential was preserved. Together, these results suggest that PBM-induced mitochondrial remodeling occurs without compromising mitochondrial functional integrity.

## Discussion

Photobiomodulation (PBM) has emerged as a promising tool in biomedical applications [20], particularly due to its ability to modulate cellular activity, enhance mitochondrial dynamics, and promote cell proliferation and differentiation. In this study, we assessed the effects of PBM on hiPSCs and analyzed its impact on cell viability, differentiation efficiency, mitochondrial function and morphology. Using 810 nm light from LEDs, we treated undifferentiated hiPSCs at three different doses. Irradiation at an intensity of 1.50 mW/cm^2^ promoted cell division and increased the proportion of cells in the S phase, suggesting that PBM promotes cell cycle progression and supports stem cell expansion. Given mitochondria as a primary site of light absorption, we also evaluated mitochondrial function through oxygen consumption rate measurements. Interestingly, although PBM enhanced hiPSC growth and promoted differentiation, basal mitochondrial respiration was not significantly upregulated. However, the mitochondrial mass and morphology differ after light treatment, suggesting that PBM may exert its effects through structural or dynamic modifications of the mitochondria [21]. These changes may further influence the maturation and functionality of cardiomyocytes [22]. During cardiomyocyte differentiation, mitochondria transition from small, fragmented structures with minimal cristae in undifferentiated cells to more elongated, networked organelles with developed cristae in mature cardiomyocytes, supporting the increased energy demands of contractile function. A more interconnected mitochondrial network potentially indicates enhanced mitochondrial function and metabolic shift from glycolysis-dependent pluripotent stem cells to oxidative phosphorylation-reliant differentiated cells.

To investigate potential pathways mediating the effects of PBM, we explored reactive oxygen species (ROS) as a candidate mechanism for regulating cellular signaling in response to environmental stimuli. Nevertheless, there was no significant difference in the ROS level between the PBM-treated and control groups in our measurement (supplementary Fig. 1), indicating that ROS are unlikely to be the primary mediator of PBM’s effects on hiPSCs. Further investigations into alternative signaling pathways [5] might be needed to better understand how PBM modulates cellular and subcellular processes in hiPSCs and cardiomyocytes.

Regarding the PBM effects on hiPSC differentiation toward cardiomyocytes, our analysis showed no difference in beating rate between the PBM group and the control group. Western blot results indicate that PBM did not substantially alter the differentiation markers of cardiomyocytes. Nevertheless, the beating activity was observed more frequently in early differentiation in the light treatment group, indicating that some underlying mechanisms affect differentiation yields and mitochondrial morphology [23]. These findings underscore the complexity of PBM’s effects on hiPSCs and their differentiation into cardiomyocytes. Further mechanistic studies and the optimization of light parameters could help maximize the beneficial effects of PBM on hiPSC differentiation and cardiomyocyte maturation.

## Supplementary Information

Below is the link to the electronic supplementary material.

Supplementary Material 1

Supplementary Figure S1 (PNG 224 KB)

Supplementary Material 2

## Data Availability

The data that support the findings of this study are available from the corresponding author upon reasonable request.
