# Supplementary material for: Enhancing human-induced pluripotent stem cell proliferation and cardiac differentiation through 810-nm photobiomodulation
Source: Lasers Med Sci. 2026 Feb 10;41(1):25. doi: 10.1007/s10103-026-04821-8 (PMC12890996; doi:10.1007/s10103-026-04821-8)
Supplement: Supplementary file 1 — Supplementary Material 1 [file 10103_2026_4821_MOESM1_ESM.docx]

Supplementary Figure


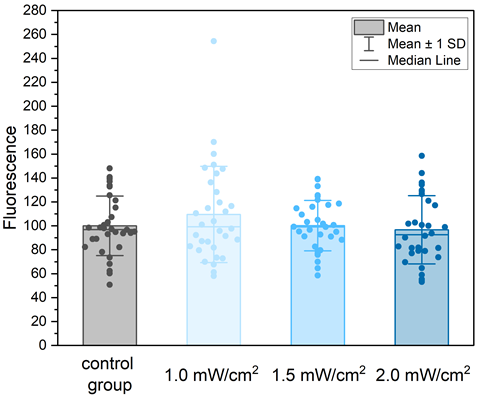


**Figure S1.** Relative ROS levels in different light treatment groups. Each data point represents cells from each well of a black 96-well plate (n = 32 for each group). ROS was monitored by CellROX DeepRed.

**Figure S2.** Quantitative analysis of mitochondrial membrane potential using TMRM fluorescence. Mitochondrial membrane potential was assessed in Control and PBM-treated groups using confocal microscopy. Image analysis was performed using a Python-based pipeline incorporating denoising, CLAHE contrast enhancement, sigmoid intensity adjustment, and Otsu thresholding to generate binary mitochondrial masks. Data are presented as the average TMRM integrated fluorescence intensity normalized to mitochondrial area (a.u./µm²). Area was calculated based on a pixel size of 0.105 µm/pixel.
