# Supplementary figures and images for: Enhancing human-induced pluripotent stem cell proliferation and cardiac differentiation through 810-nm photobiomodulation
Source: Lasers Med Sci. 2026 Feb 10;41(1):25. doi: 10.1007/s10103-026-04821-8 (PMC12890996; doi:10.1007/s10103-026-04821-8)

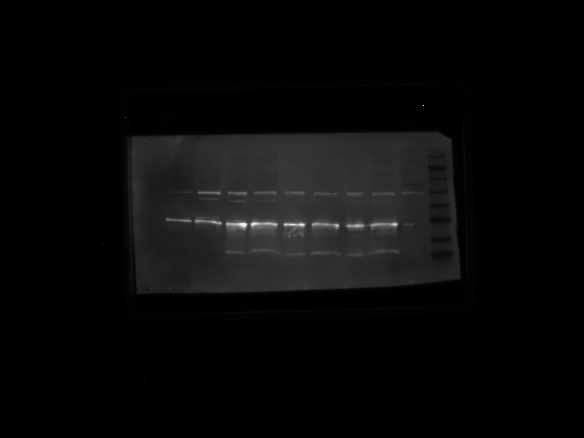

Supplement: Supplementary file 2 — (PNG 224 KB) [file 10103_2026_4821_Fig7_ESM.png]

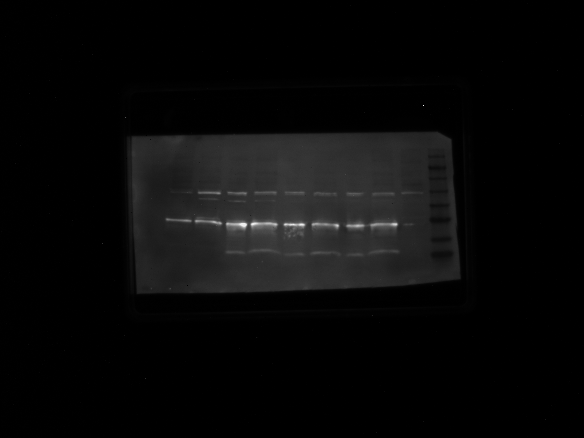

Supplement: Supplementary file 3 — Supplementary Material 2 [file 10103_2026_4821_MOESM2_ESM.tif]
